# Healthy subjects gut microbiome modulation by *Bacillus coagulans* BCP92: A randomized, double-blind, placebo-controlled clinical trial

**DOI:** 10.1097/MD.0000000000050435

**Published:** 2026-08-28

**Authors:** Sohel S. Shaikh, Farhana Malek

**Affiliations:** a Pellucid Lifesciences Pvt Ltd, Gandhinagar, Gujarat, India.

**Keywords:** *Bacillus coagulans* BCP92, Gut microbiome, Healthy Subject, Metagenome analysis, Probiotic, Short-chain fatty acids

## Abstract

**Background:**

Probiotics are recognized for their ability to restore balance in the gut microbiome during dysbiosis. However, their effects on the gut microbiota of healthy individuals have rarely been investigated. This study aimed to evaluate the safety and efficacy of *Bacillus coagulans (Heyndrickxia coagulans*) BCP92 and its influence on microbiota composition in healthy subjects.

**Methods:**

In the present investigation, healthy participants (n = 48) were allocated into 2 groups and administered either *Bacillus coagulans* BCP92 capsules (1 billion CFU/capsule) or a placebo containing maltodextrin for 42 days. Microbiome composition and short-chain fatty acid analyses were subsequently conducted.

**Results:**

Analysis of metagenomes showed no major alterations in gut microbiome composition among participants who received *B. coagulans* BCP92 supplementation. However, subtle beneficial changes were observed in the treatment group, suggesting that probiotic administration may increase advantageous phyla, classes, orders, families, and some genera, while decreasing potentially harmful groups. A slight increase in short-chain fatty acids (SCFA) was also observed in the fecal samples.

**Conclusions:**

This study implies that extended supplementation with the probiotic *B. coagulans* BCP92 may lead to substantial improvements in gut microbiome composition and SCFA levels.

## 1. Introduction

The gut microbiome plays a vital role in various metabolic processes and functions, including control of intestinal epithelial cell growth,^[1]^ protection against colonization by harmful organisms,^[2]^ and regulation of immune responses in the intestines.^[3]^ The human intestinal region harbors more than 1500 bacterial species that colonize immediately following birth, surpassing the microbial diversity of any other anatomical region. This bacterial diversity establishes a mutually beneficial or symbiotic relationship with epithelial and lymphoid tissues.^[4]^

The human body and the gut microbiota operate in a symbiotic relationship, contributing to overall health through metabolic, protective, and structural functions.^[5]^ Consequently, alterations in an individual’s characteristic gut microbiome composition may result in an increased susceptibility to various health conditions.^[6–10]^ Lifestyle and diet affect the balance of intestinal microbiome composition.^[11,12]^ Nevertheless, microbiota dysbiosis has been reported in various pathological conditions, including gut inflammatory disorders, cardiovascular diseases, autoimmune disorders, and respiratory disorders.^[13–15]^ Moreover, common treatments such as antibiotics, which are often prescribed to combat infections, unintentionally eliminate helpful bacteria. This process disrupts the balance between harmful and nonharmful microorganisms.^[16,17]^

The prevalence of probiotics has been increasing, and probiotics are typically marketed as functional foods or nutritional supplements. Owing to the established role of gut microbiota alterations in gastrointestinal disorders, the use of probiotics has shown potential as a promising method for modulating the microbial balance in the gut.^[18]^

*Bacillus coagulans* BCP92 has been demonstrated to be a nonpathogenic, safe^[19,20]^ strain of *Bacillus* that remains viable during storage before being consumed, as the spores exhibit thermostability, remain stable in gastric secretion, and produce lactic acid. It is reported to be stable under various food storage and processing conditions^[21]^ and is effective in irritable bowel syndrome (IBS).^[22]^ In the present study, we studied the impact of *B. coagulans* BCP92 supplementation on the gut microbiome, short-chain fatty acid levels, blood and urine parameters to assess safety. Furthermore, the effect of *B. coagulans* BCP92 consumption on vital signs was examined in healthy individuals.

## 2. Methods and materials

### 2.1. Study design and ethics

This was a randomized, double-blind, placebo-controlled clinical trial with two arms to assess the safety and effectiveness of the probiotic *Bacillus coagulans* BCP92 in healthy adults. The investigation was conducted at Cliantha Research in Ahmedabad. After obtaining written informed consent, potential participants were evaluated based on the specific inclusion and exclusion criteria. The study protocol (C3B04178), including the Informed Consent Document (ICD), was examined and approved by the ACEAS – Independent Ethics Committee, adhering to the Indian Council of Medical Research (ICMR) Ethical Guidelines, International Council for Harmonization of Technical Requirements of Pharmaceuticals for Human Use – Good Clinical Practice (ICH-GCP), New Drugs and Clinical Trials Rules, 2019, and the Declaration of Helsinki. This trial was registered in the Clinical Trial Registry of India (CTRI) on April 10, 2024 (registration number: CTRI/2024/04/066125).

### 2.2. Randomization and blinding

A biostatistician prepared the randomization schedule. A randomization schedule was generated using SAS® software (Version 9.4; SAS Institute Inc., USA) to ensure balanced treatment allocation. The randomization schedule was maintained under restricted access conditions. Personnel responsible for dispensing the test products were accountable for ensuring adherence to the randomization schedule. The participants and clinical staff were blinded to the trial group assignments.

### 2.3. Study population

A total of 48 healthy adult subjects were enrolled, of whom 43 completed the study. The study included participants who fulfilled all of the following requirements: individuals between 18 and 55 years old at the time of providing consent and male and female volunteers in good overall health, with females not being pregnant or breastfeeding. The Investigator assessed the participants’ health status through a review of their medical history, physical examination, and measurement of vital signs (i.e., respiration rate, pulse rate, and blood pressure). Individuals who agreed to abstain from consuming any probiotic food or supplement, including yogurt/buttermilk, during the course of this study; those who agreed to refrain from consuming pharmaceuticals, such as anti-inflammatory, immunosuppressive, and laxative agents; and those who were willing to maintain a consistent diet and lifestyle routine throughout the study.

The exclusion criteria included a history of acute peptic ulcer, severe gastroesophageal reflux disease (GERD) and/or ulcer complications, or other functional gastrointestinal disorders. Patients with any pathological condition associated with gastroesophageal reflux disease, including Barrett’s esophagus or malignancy, were excluded. The study also excluded Subjects with chronic illnesses or health conditions such as cancer, diabetes, Peripheral Artery Disease, Chronic kidney disease (CKD), previous major surgery, human immunodeficiency virus (HIV), hepatitis, and other similar conditions. Furthermore, participants with diseases such as hypothyroidism, diabetes, Parkinson’s disease, Alzheimer’s disease, and dementia, were not included. Participants with ongoing medical or psychiatric conditions that could jeopardize their safety, impact the validity of the study results, or interfere with the completion of the study according to the protocol were also excluded. The exclusion criteria were extended to individuals who regularly used medications affecting bowel habits, such as those for irritable bowel syndrome, functional bloating, and functional diarrhea. Subjects who had consumed probiotics or prebiotics within 30 days before the study were excluded. Subjects with known allergies, hypersensitivity to probiotics, and/or any ingredients in the test product were excluded. Finally, participants with a history or presence of significant alcoholism, drug abuse, smoking, or consumption of tobacco products were excluded from the study. Enrollment began in May 2024, and the treatment of the last subject was completed in June 2024.

### 2.4. Interventions

Probiotic investigational product (IP) and placebo were provided by Pellucid Lifesciences PVT. Ltd. Each capsule of the probiotic *Bacillus coagulans* BCP92 contained 1 billion colony-forming units (CFU), and the placebo contained maltodextrin. The participants were instructed to consume one capsule of the test or placebo product with water for 42 days.

### 2.5. Endpoints and outcomes

#### 2.5.1. Metagenomic analysis of gut microbiome

For microbiome analysis, stool specimens were obtained during the initial and final visits using designated fecal collection containers. Genomic DNA was extracted from each sample utilizing a Qiagen gDNA kit. The quality of the extracted gDNA was evaluated by running 5.0 μl on 0.8% agarose gel at 110 V for 30 minutes and observing a single intact band. A BioTeK Epoch device was used to measure the A260/280 ratio using 2.0 µl of each sample. DNA was quantified using the Qubit dsDNA HS Assay Kit (Life Technologies). A Qubit^®^ 2.0 Fluorometer was used to determine the concentration of each sample, utilizing 1.0µl for measurement.

Prokaryotic 16S rDNA was amplified using Ion AmpliSeq™ Microbiome Health Research Kits (Catalog Numbers A46496 and A46497; Thermo Fisher Scientific, MA, USA). These kits contain 16S rRNA gene primer sets that amplify eight hypervariable regions (HV2–HV9) of the bacterial 16S region. Following amplification, adapters and barcodes were attached to the amplicons and purified using the Agencourt AMPure XP reagent (Beckman Coulter, CA, USA) on a magnetic rack, according to the manufacturer’s protocol. The libraries were then loaded onto a 550 chip and sequenced using the Ion Torrent S5 Plus system (Thermo Fisher Scientific, MA, USA). A minimum of 14.4 ng of total DNA was used for 16S library preparation, and re-amplification was performed using the Ion Plus Fragment Library kit to achieve the required template concentration. An equimolar pool of libraries was evaluated using an Agilent Bioanalyzer 2100 with a DNA1000 chip (Agilent Technologies, CA, USA). Alpha diversity was assessed using Kraken2, whereas beta diversity was assessed using QIIME 2. The abundance of species in the metagenomic samples was determined using Bracken software.^[23]^

#### 2.5.2. Short chain fatty acid analysis

An Agilent Technologies 1260 Infinity Quaternary High-performance liquid chromatography (HPLC) system was used to quantify the concentrations of short-chain fatty acids (SCFAs), specifically butyrate, acetate, and propionate, in human fecal samples. The analysis was conducted using a 100 µL loop auto-sampler controlled by OpenLAB CDS EZChrom software (version A.01.03). To prepare the samples, 100 mg of each fecal specimen was mixed with 1 mL of HPLC-grade water and thoroughly blended. The resulting mixture was centrifuged at 10,000 rpm for 15 minutes. The obtained supernatant was then diluted 200 times and analyzed using HPLC. Chromatographic separation was achieved using a mobile phase consisting of 0.05% phosphoric acid (H_3_PO_4_) buffer (pH 3.5) and acetonitrile in a 98:2 ratio. An Agilent Zorbax SB-CN column (5 µm, 4.6 × 250 mm) was employed and maintained at 30°C with a flow rate of 1 mL/min. Detection was performed at 210 nm using an injection volume of 20 µL. The quantity of SCFAs was determined by comparing the retention times and peak areas of the butyric, propionic, and acetic acids with those of their corresponding reference solutions.

### 2.6. Safety assessment

To evaluate the impact of products on Liver Function tests (Serum Glutamic Pyruvic Transaminase (SGPT) and Serum Glutamic-Oxaloacetic Transaminase (SGOT)), Renal Function Tests (Blood Urea Nitrogen (BUN), Serum Creatinine, and Serum Uric Acid), Random Blood glucose, thyroid function tests (triiodothyronine (T3), thyroxine (T4), thyroid-stimulating hormone (TSH), Complete Blood Count (CBC), Red Blood Cells (RBC), white blood cells (WBC), Platelets, Hemoglobin and Hematocrit, and urine tests (Urine Routine & Microscopy).

### 2.7. Statistical analysis

To analyze categorical variables, change from baseline (CFB), and % CFB paired *t*-test was used. A two-sample *t*-test was used to compare the 2 products. Statistical significance was set at a threshold of *P *< .05. The Bracken method was used for metagenomic analysis. Statistical significance was assessed using an unadjusted two-sided *P*-value threshold of < .05. No adjustment for multiple comparisons was performed because the analyses were considered exploratory. GraphPad Prism 9.1.0 software was utilized for statistical analysis. Analyses were conducted on a per-protocol basis, including only participants who completed the study.

## 3. Results

The study initially enrolled 48 of the 53 screened individuals. However, 5 participants could not complete the study because of missed follow-up appointments. Consequently, the final analysis was based on the data from the remaining 43 participants who completed the entire study protocol. The participants’ dispositions throughout the study are presented in Table 1, and their demographics are presented in Table 2.

**Table 1:** Disposition of study subjects.

Description	Count (Percent)
Screened subjects	53 (100%)
Screening fail subject	5 (9.43%)
No. of Subject Screen pass but not enrolled	0 (0%)
Randomized/Enrolled subjects	48 (90.57%)
Completed subjects	43 (89.58%)
Discontinued subjects/ withdrawal	5 (10.42%)
Premature termination	NA
Reason for Discontinued subjects/withdrawal	Lost to follow up

**Table 2:** Demographic and other baseline characteristics.

Summary of Demographic Characteristics					
Variables		Code A (IP) (N = 24)	Code B (Placebo) (N = 24)	Overall (N = 48)	*p* value
Race n (%)	Asian	24 (100%)	24 (100%)	48 (100%)	^_^
Gender n (%)	Female	11 (45.83%)	9 (37.50%)	20 (41.67%)	
	Male	13 (54.17%)	15 (62.50%)	28 (58.33%)	.7701^X2^
Age (Years)	n	24	24	48	
	Mean ± SD	36.6 ± 9.51	40.3 ± 8.08	38.4 ± 8.92	.1682*
	Median	35.5	40.5	38.5	
	Min, Max	21, 51	26, 54	21, 54	

SD = standard deviation, *P* < .05 shows statistical significance, X^2^ - chi square test, *Two-sample *t* test.

In this study, there were 28 males and 20 females; age of the participants ranged from 21 to 54 years with average being 38.4 years.

### 3.1. Metagenomic analysis of gut microbiome

**Figure 1.**
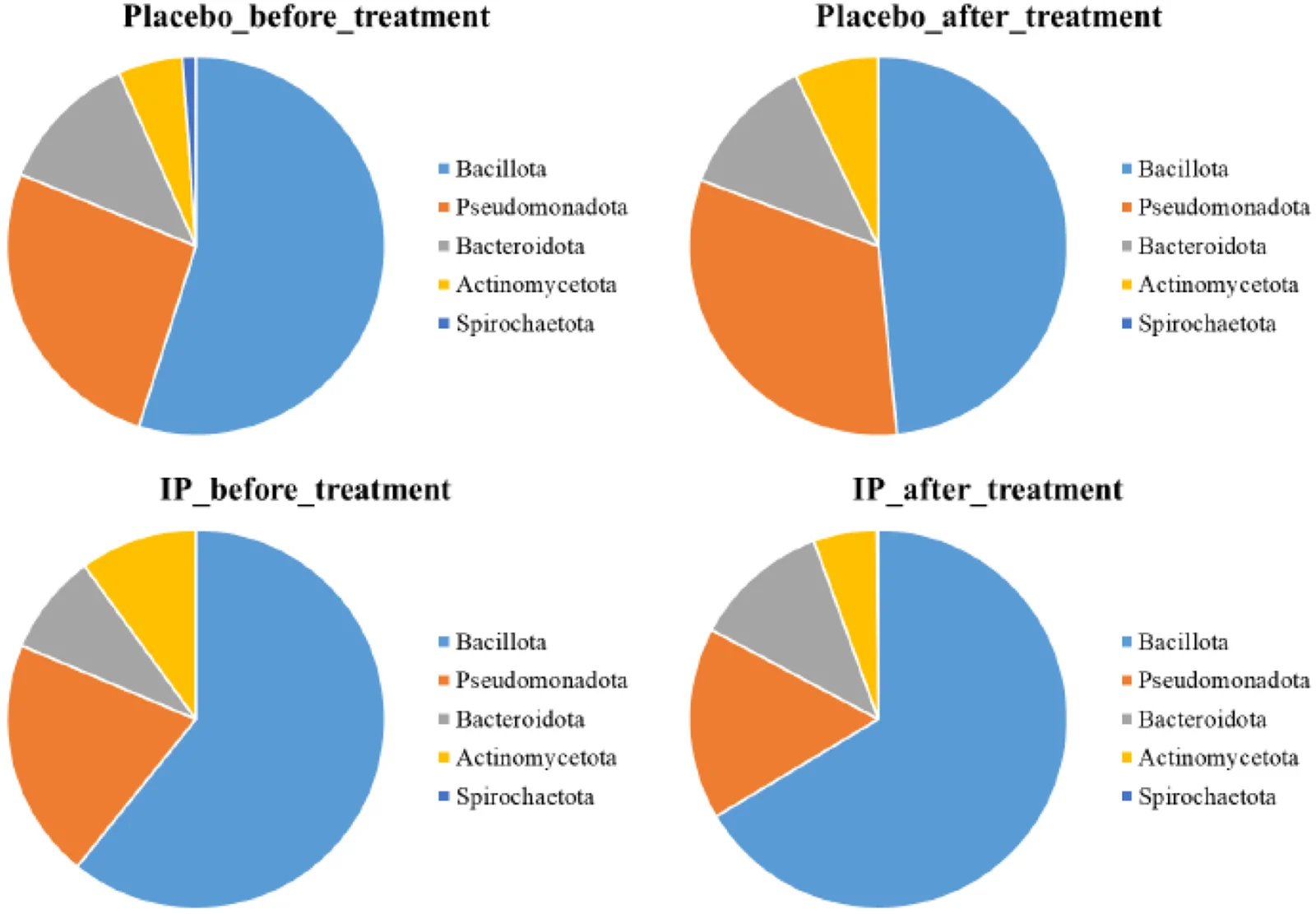
Comparison of microbial Phylum abundance before and after treatment in the placebo and probiotic (IP) groups, IP = *B. coagulans* BCP92.

Bracken, a highly precise statistical technique, determines microbial abundance in metagenomic DNA samples. At the phylum level, findings indicated that Bacillota, a phylum containing beneficial bacteria, exhibited a significant increase in the IP group (from 60.62% to 65.81%) while declining in the placebo group (from 54.69% to 47.80%), suggesting a favorable effect of the probiotic. Conversely, Pseudomonadota, which is associated with potentially harmful bacteria, increased in the placebo group (from 26.18% to 31.76%) but decreased in the IP group (from 20.48% to 16.24%), indicating its potential role in inhibiting this phylum. Bacteroidota, crucial for carbohydrate metabolism, remained constant in the placebo group (from 12.12% to 12.00%) but demonstrated a minor increase in the IP group (from 8.60% to 11.55%), suggesting a subtle advantage of the probiotic in supporting this phylum. Actinomycetota increased in the placebo group (from 5.50% to 7.07%) but decreased in the IP group (from 9.99% to 5.34%), potentially indicating the probiotic’s selective influence on the microbial composition. Spirochaetota, a minor phylum, was nearly eliminated in the placebo group but persisted at low levels in the IP group. The other category, comprising less prevalent phyla, increased slightly in both groups (Fig. 1).

**Figure 2.**
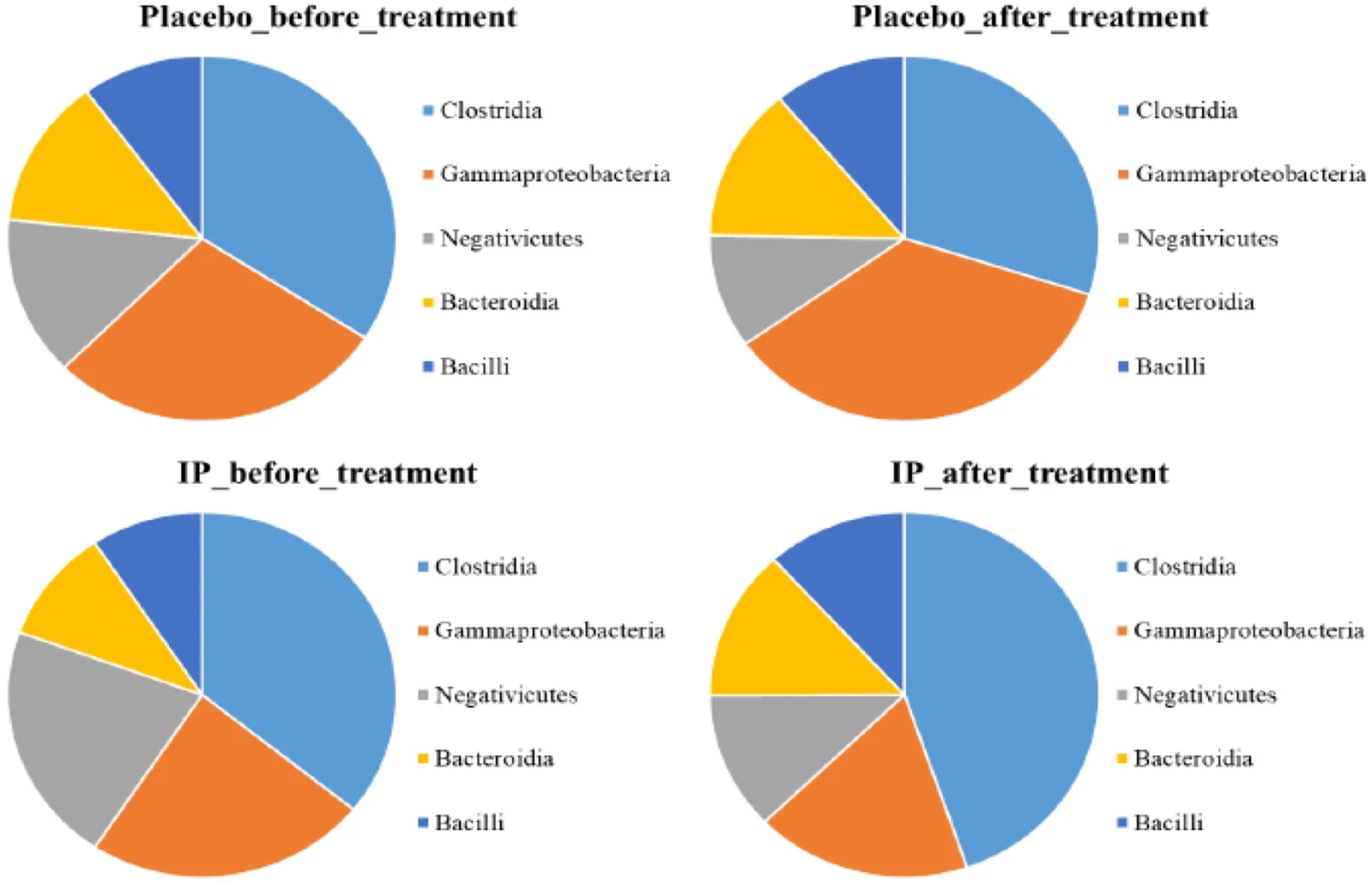
Comparison of microbial Class abundance before and after treatment in the placebo and probiotic (IP) groups, IP = *B. coagulans* BCP92.

The class-level results showed that Clostridia, a class associated with beneficial gut functions, increased in the IP group (from 30.22% to 38.55%) but decreased in the placebo group (from 30.76% to 25.82%), suggesting the probiotic’s role in supporting this class. Gammaproteobacteria, which can be linked to inflammation, decreased in the IP group (from 19.82% to 15.48%) but increased in the placebo group (from 25.51% to 30.36%), indicating that probiotics may help reduce potentially harmful taxa. Negativicutes decreased in both groups, but more so in the placebo group, which could indicate a natural reduction unrelated to probiotics. Bacteroidia, which are important for fiber metabolism, showed stability across both groups, with a slight increase in the IP group (from 8.53% to 11.50%). The number of bacilli, which are often beneficial for gut health, increased in both groups, with a higher posttreatment percentage in the IP group (from 7.87% to 10.09%). The other category, representing less abundant classes, increased in the placebo group but remained stable in the IP group (Fig. 2).

**Figure 3.**
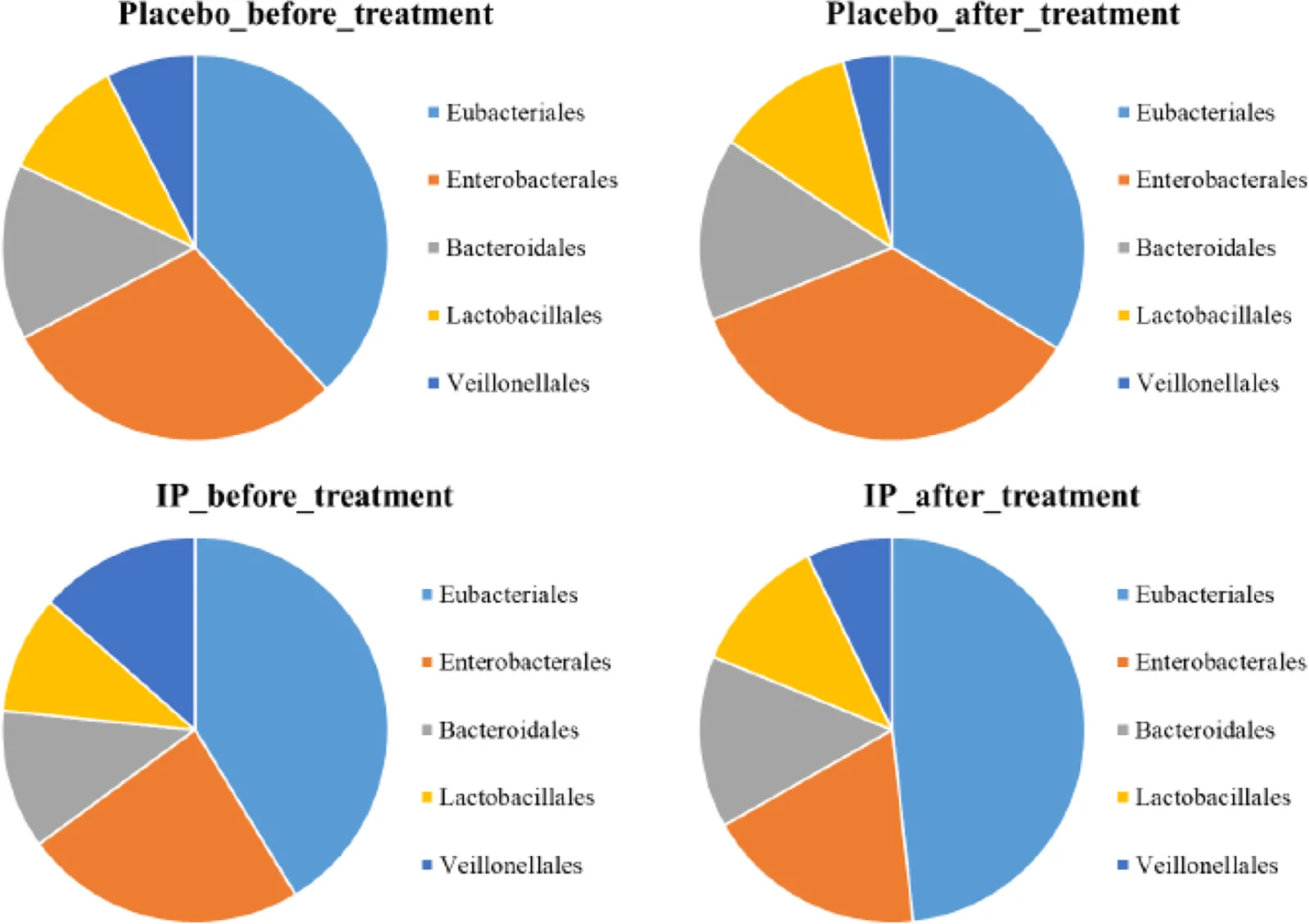
Comparison of microbial Order abundance before and after treatment in the placebo and probiotic (IP) groups, IP = *B. coagulans* BCP92.

Analysis at the order level revealed that Eubacteriales, a beneficial order, exhibited a significant increase in the IP group (from 30.21% to 38.54%) and a decrease in the placebo group (from 30.75% to 25.81%), suggesting that probiotics may enhance the presence of this order. Enterobacterales, which encompasses potentially pathogenic bacteria, increased in the placebo group (from 23.60% to 26.98%) but decreased in the IP group (from 17.31% to 14.78%), indicating a possible suppressive effect of the probiotic on potentially harmful bacteria. Bacteroidales, involved in carbohydrate metabolism, remained relatively stable in the placebo group (from 11.90% to 11.73%) but increased in the IP group (from 8.53% to 11.50%), suggesting a positive response to the probiotic. Lactobacillales, associated with beneficial gut bacteria, showed a slight increase in both groups, with a more pronounced increase in the IP group (from 7.25% to 9.28%), indicating enhanced growth potentially attributable to probiotics. Veillonellales, which are potentially associated with inflammation, decreased in both groups, placebo (from 6.04% to 3.12%), with a more substantial reduction observed in the IP group (from 9.88% to 5.75%), suggesting probiotic modulation. The prevalence of less common bacterial orders, grouped under Other, showed contrasting trends between the placebo and IP groups. Although this category increased in the placebo group, it decreased in the IP group, suggesting that probiotic supplementation led to a more equilibrated microbial community structure (Fig. 3).

**Figure 4.**
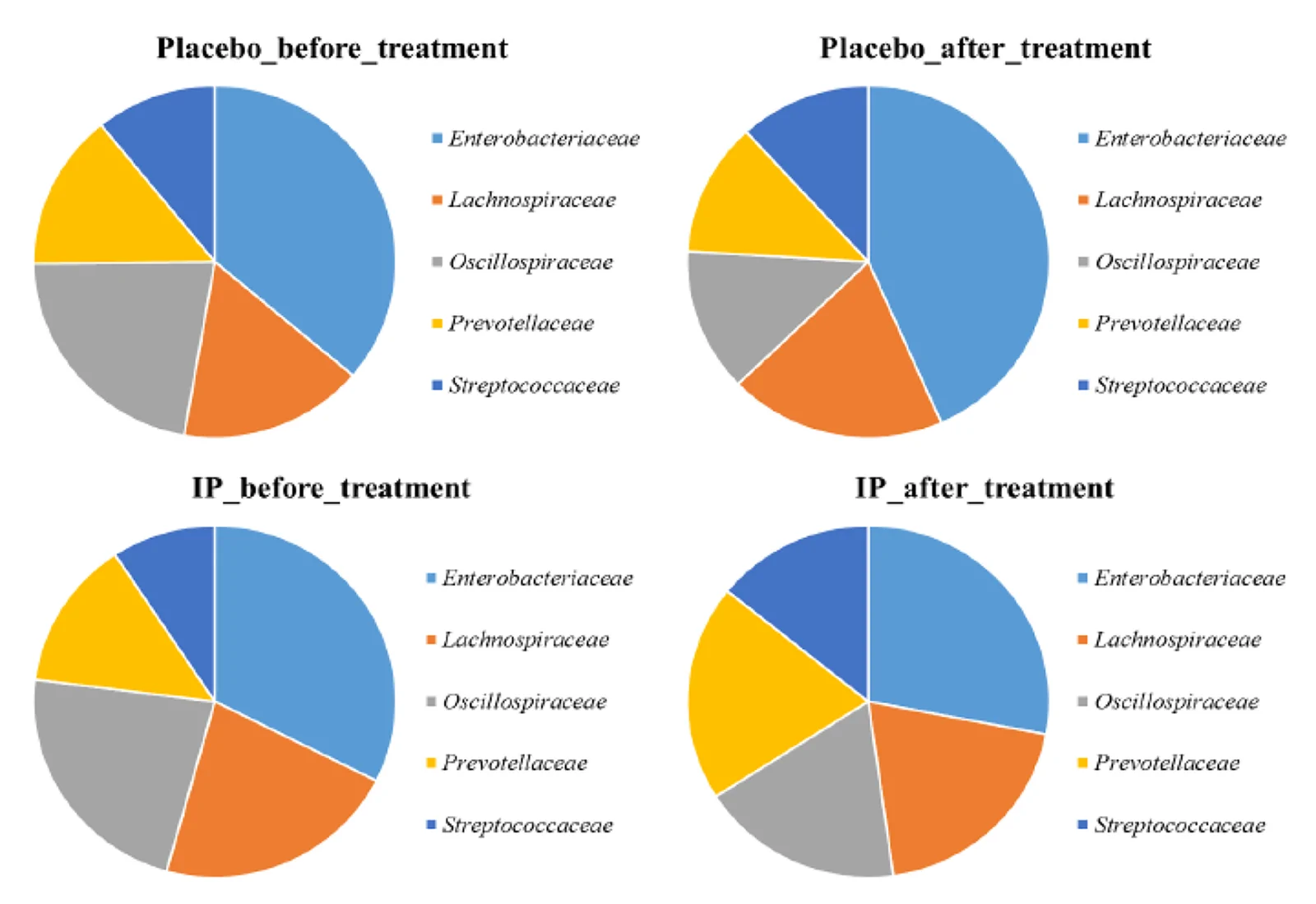
Comparison of microbial Family abundance before and after treatment in the placebo and probiotic (IP) groups, IP = *B. coagulans* BCP92.

Family-level analysis results indicated that Enterobacteriaceae, which encompasses potentially pathogenic bacteria, increased in the placebo group (from 23.26% to 26.54%) but decreased in the IP group (from 17.07% to 14.53%), suggesting a potential suppressive effect of probiotics. Lachnospiraceae, known for producing beneficial short-chain fatty acids, exhibited a slight increase in the placebo group (from 10.62 to 11.87%) but a slight decrease in the IP group (from 11.49% to 10.26%), possibly due to probiotic-mediated modulation. Oscillospiraceae, another beneficial family, demonstrated a notable reduction in both groups, although the decrease was less pronounced in the IP group, indicating potential probiotic support for stabilizing this family. Prevotellaceae, which is associated with carbohydrate metabolism, decreased in the placebo group (from 9.23% to 7.52%) but increased in the IP group (from 13.28% to 14.18%), suggesting that probiotics may favorably influence this family. Streptococcaceae, which contains beneficial strains, exhibited a mild increase in both groups, with a greater increase in the IP group (from 4.89% to 7.37%), indicating potential probiotic benefits. The other category, representing less prominent families, increased in both groups, with a slightly higher increase observed in the IP group. Overall, these results suggest that probiotics may help reduce potentially harmful families, such as Enterobacteriaceae, while promoting beneficial families, such as Prevotellaceae and Streptococcaceae, thereby supporting a healthy gut microbiome balance (Fig. 4).

A genus-level analysis of the gut microbiota was conducted to evaluate the effect of probiotic supplementation compared with placebo over the study period. Changes in the bacterial genera were assessed before and after treatment in both groups.

The results from the IP group indicated a positive modulation in several bacterial genera, including *Bifidobacterium*, an important genus known for its immunomodulatory functions, which increased (from 6.97% to 7.32%). *Bacteroides*, involved in the production of Short-Chain Fatty Acids (SCFAs), increased (from 1.18% to 2.31%). *Prevotella*, linked to fiber metabolism, increased slightly (from 7.21% to 10.31%). *Faecalibacterium* decreased moderately (from 11.44% to 6.43%). However, this reduction was lower than that observed in the placebo group.

*Ruminococcus, Catenibacterium*, and *Streptococcus* exhibited increased relative abundance, increasing (from 1.00% to 2.34%), (from 2.50% to 3.48%), and (from 1.78% to 3.03%), respectively. In contrast, the abundances of *Megasphaera, Lactobacillus,* and *Roseburia* decreased (from 5.05% to 3.62%), (from 2.07% to 1.45%), and (from 2.59% to 1.45%), respectively. The proportion of other genera remained stable, changing slightly (from 43.72% to 42.83%).

**Figure 5.**
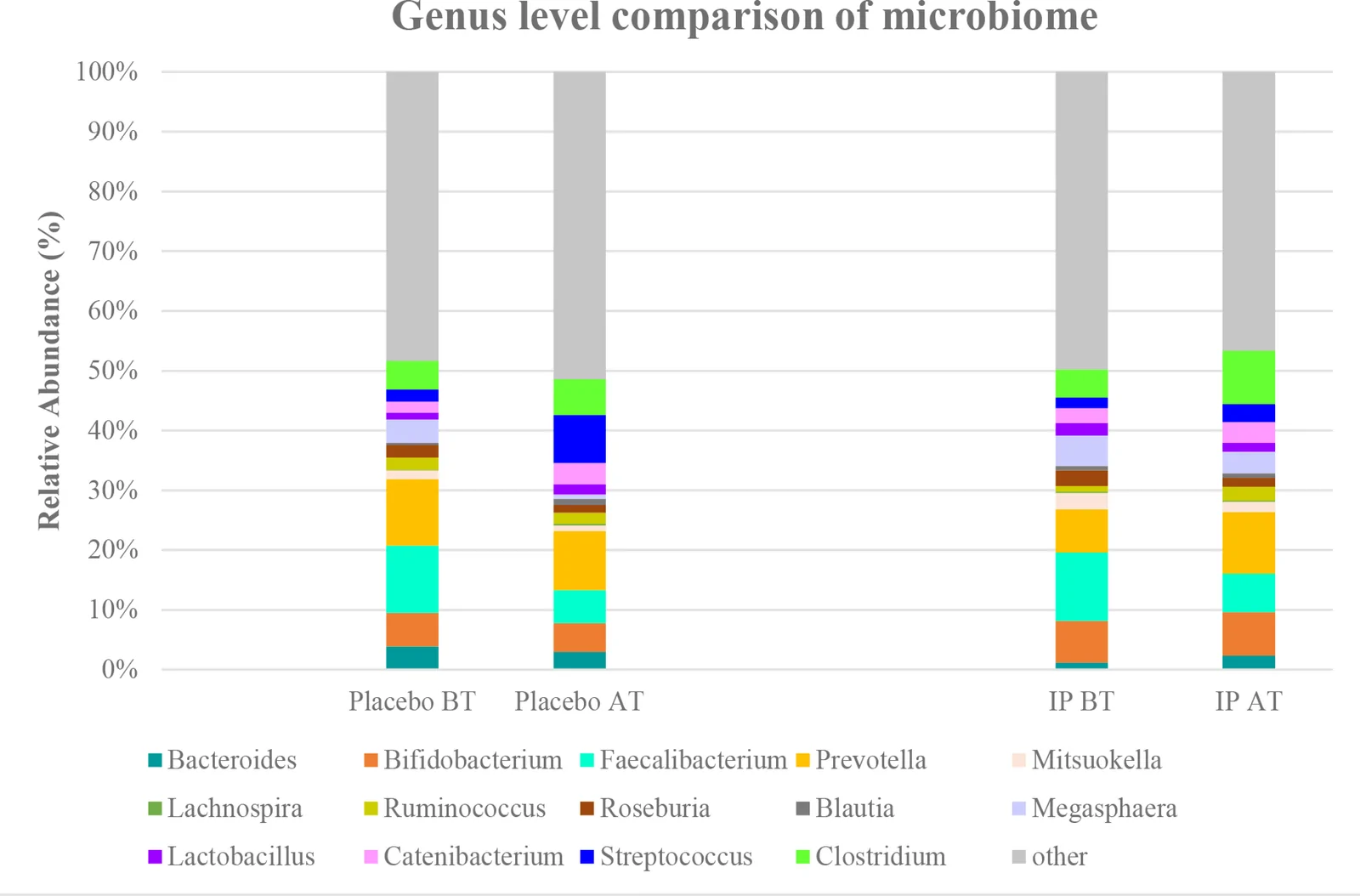
Comparison of microbial genus abundance before and after treatment in the placebo and probiotic (IP) groups. BT = Before treatment, AT = after treatment, IP = *B. coagulans* BCP92.

The placebo group showed an increase in the relative abundance of *Lactobacillus* (from 1.16% to 1.73%), *Catenibacterium* (from 1.85% to 3.54%), *Blautia* (from 0.39% to 1.06%), *Lachnospira* (from 0.07% to 0.26%), and *Streptococcus* (from 2.04% to 7.99%). The relative abundance of the genus *Bacteroides* (from 3.86% to 2.92%), *Bifidobacterium* (from 5.64% to 4.86%), and *Faecalibacterium* (from 11.22% to 5.48%) decreased (Fig. 5).

#### 3.1.1. Alpha diversity

Measures of alpha diversity offer insights into the intricacy of microbial ecosystems within individual samples. These metrics evaluate both richness (total species count) and evenness (how uniformly individuals are distributed among species) of a community. Various indices have been used to capture the distinct aspects of diversity within these microbial populations.

##### 3.1.1.1. Shannon’s diversity index

**Figure 6.**
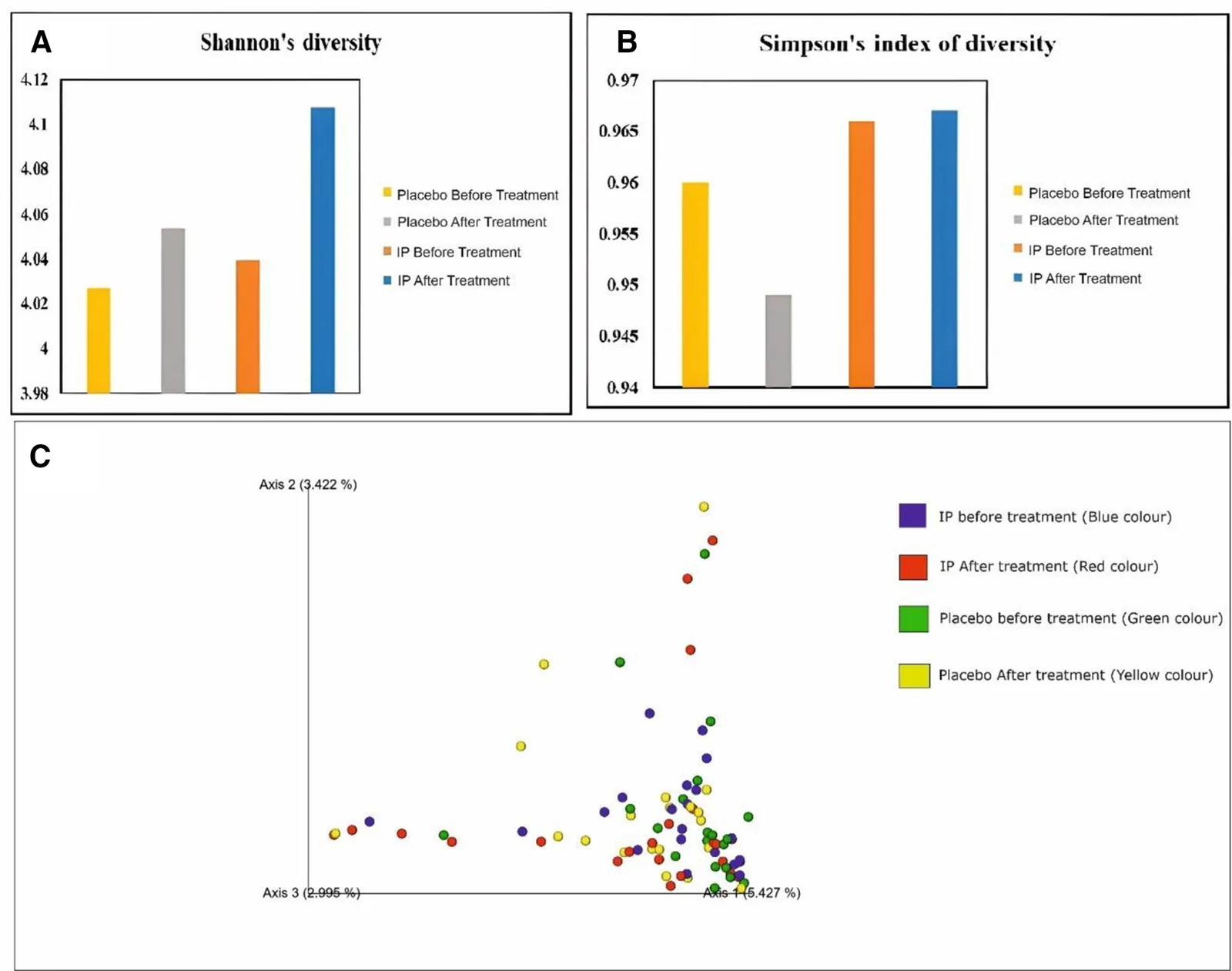
Microbial diversity and composition analysis across placebo and probiotic (IP) groups. (A) Shannon’s diversity index (B), Simpson’s diversity index, and (C) Principal Coordinates Analysis (PCoA) plot showing beta diversity, highlighting differences in microbial community composition among samples.

This index considers both the richness (number of species) and evenness (species distribution). Higher values indicate greater diversity. A higher index value indicates greater biodiversity. In the IP group, Shannon’s index increased from 4.039 to 4.107 posttreatment, indicating a modest increase in species diversity. Conversely, the Placebo group exhibited only a marginal change, with the index moving from 4.027 before treatment to 4.054 after treatment, suggesting that the placebo had little impact on species diversity (Fig. 6).

##### 3.1.1.2. Simpson’s index of diversity

Values approaching one for this index suggest a higher level of diversity. The IP group exhibited a consistently high value, shifting only slightly from 0.966 to 0.967 pre- and posttreatment, respectively, indicating sustained diversity. In contrast, the placebo group showed a minor decline from 0.96 to 0.949 after treatment, suggesting a small decrease in diversity (Fig. 6).

#### 3.1.2. Beta diversity

To examine variations in microbial community composition among the samples, we employed principal coordinate analysis (PCoA) based on Bray-Curtis dissimilarity measures.

This Principal Coordinates Analysis (PCoA) plot provides a beta diversity visualization of the differences in microbial community composition among samples, wherein proximal points represent more similar communities and those that are more distant are more dissimilar. Each color-coded point represents a sample group based on treatment and timing: IP after treatment (red) exhibits a distinct microbial composition compared to IP before treatment (blue), placebo after treatment (yellow), and placebo before treatment (green), indicating that IP treatment slightly affects microbial composition compared to the baseline and placebo. Overall, the results showed slight variation in the microbial communities (Fig. 6).

### 3.2. Short-chain fatty acid analysis

**Figure 7.**
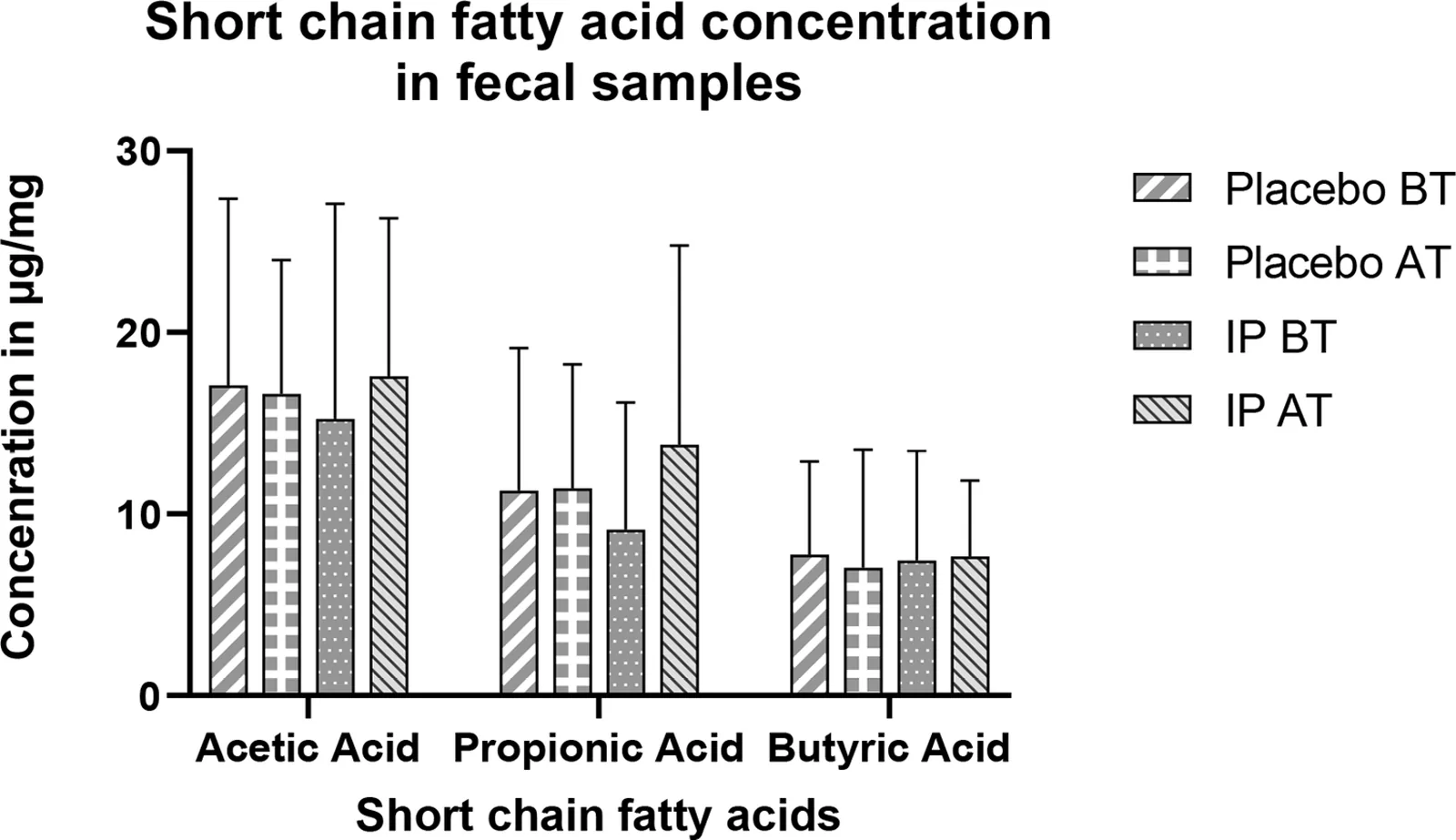
SCFA concentrations (mean ± SD) in fecal samples for Placebo BT, Placebo AT, IP BT, and IP AT groups. Placebo BT (Before Treatment), Placebo AT (After Treatment), IP BT (Investigational Product Before Treatment), and IP AT (Investigational Product After Treatment).

Substantial changes in SCFA concentrations were observed between the IP-Before Treatment (IP-BT) and IP-After Treatment (IP-AT) conditions. The mean acetic acid level increased from 15.24 ± 11.84 to 17.59 ± 8.69, although this change was not statistically significant (N = 23, *P = .4465*). Propionic acid levels also increased from 9.14 ± 7.00 to 13.81 ± 10.97; however, this increase was not statistically significant (*P = .0923*). Butyric acid levels showed a marginal increase, from 7.44 ± 6.02 to 7.66 ± 4.17 (*P = .8831*). The concentrations of short-chain fatty acids (SCFAs) showed minimal variation Before Treatment (Placebo BT) and After Treatment (Placebo AT). The mean acetic acid levels slightly decreased from 17.08 ± 10.29 to 16.61 ± 7.38 (*N* = 20, *P = .8701*). Propionic acid levels remained nearly unchanged, with a mean of 11.30 ± 7.84 before treatment and 11.42 ± 6.83 after treatment (*P = .9592*). Similarly, butyric acid levels showed a slight reduction from 7.76 ± 5.13 to 7.03 ± 6.52 (*P = *.6977) (Fig. 7).

### 3.3. Safety evaluation

No local intolerance or adverse effects were observed in any of the participants during the study period, confirming the safety of the test products. No adverse events were reported or observed during the study. All vital and blood laboratory parameters [Liver Function tests (SGPT and SGOT), Renal Function Tests (BUN, Serum Creatinine, and Serum Uric Acid), Random Blood glucose, thyroid function tests (T3, T4, and TSH), CBC (RBC, WBC, Platelets, Hemoglobin and Hematocrit) (Table 3), and urine tests (Urine Routine & Microscopy)] [Tables S1 and S2, Supplemental Digital Content 1] remained within normal ranges at baseline and at the end of treatment, suggesting the absence of adverse effects from the treatment. Furthermore, all safety parameters remained within clinically acceptable ranges, with no clinically meaningful changes observed during the study. No adverse effects attributable to the intervention were observed.

**Table 3:** Results of hematological and biochemical parameters at baseline and end of study in IP and placebo groups.

Parameter	IP			Placebo			*P* Value (IP vs Placebo)
	Baseline	End of Study	*p* Value	Baseline	End of Study	*p* Value	
Total RBC Count (10^12/L)	4.50 ± 0.62	4.62 ± 0.55	.0510	4.63 ± 0.49	4.76 ± 0.40	.0683	.9642
Total WBC Count (10^9/L)	7.48 ± 2.25	7.44 ± 2.19	.8850	7.50 ± 1.48	7.61 ± 1.48	.7263	.7125
Platelets (10^9/L)	364.82 ± 14.17	330.30 ± 87.20	.1687	348.95 ± 15.01	322.65 ± 67.53	.4014	.8325
Hemoglobin (g/dL)	12.12 ± 1.76	12.41 ± 1.52	.0250*	11.49 ± 1.08	11.72 ± 1.13	.1472	.7515
Hematocrit (%)	38.23 ± 4.65	38.65 ± 3.76	.3595	36.86 ± 2.80	37.28 ± 3.00	.3524	.9862
SGPT (U/L)	19.85 ± 8.65	19.89 ± 10.28	.9704	22.61 ± 13.70	20.80 ± 8.40	.4869	.5118
SGOT (U/L)	20.88 ± 5.54	23.70 ± 10.53	.1443	24.79 ± 13.31	23.84 ± 7.01	.6909	.2092
BUN (mg/dL)	7.79 ± 2.15	7.53 ± 2.84	.6584	7.84 ± 2.82	7.91 ± 2.60	.8917	.6760
Serum Creatinine (mg/dL)	0.81 ± 0.21	0.78 ± 0.21	.0966	0.75 ± 0.15	0.76 ± 0.16	.3394	.0634
Serum Uric Acid (mg/dL)	4.92 ± 1.28	4.87 ± 1.27	.6404	4.64 ± 1.35	4.90 ± 1.45	.0499*	.0554
T3 (ug/L)	1.08 ± 0.23	1.01 ± 0.15	.0890	1.07 ± 0.13	1.03 ± 0.12	.0759	.6239
T4 (ug/dL)	7.65 ± 1.52	7.85 ± 1.73	.2641	7.50 ± 1.11	7.87 ± 1.51	.1996	.6077
TSH (uIU/mL)	2.47 ± 1.56	2.45 ± 1.51	.9358	2.86 ± 1.52	2.92 ± 2.00	.8163	.8187
Blood glucose (mg/dL)	84.54 ± 16.88	81.34 ± 14.47	.4423	87.86 ± 13.00	85.17 ± 18.69	.3037	.9175

* = Statistically Significant.

## 4. Discussion

This study aimed to assess the safety and effectiveness of *Bacillus coagulans* BCP92 in healthy adults, focusing on its impact on the gut microbiome. In addition, laboratory parameters were examined to evaluate its safety.

This study initially screened 53 individuals. Among these, 48 satisfied the inclusion criteria and were enrolled, while 5 did not meet the eligibility criteria. Throughout the course of the investigation, 5 participants were missed during follow-up, resulting in a final sample of 43 subjects who completed the study. The analysis was conducted using the data from 43 participants who completed the study protocol.

Analysis of the metagenome showed no major alterations in the gut microbiome composition among participants who received *B. coagulans* BCP92 supplementation. Subtle beneficial effects were observed in all treatment groups. Collectively, these findings indicate that probiotic administration may enhance beneficial phyla, classes, orders, and families while reducing potentially detrimental groups, thereby promoting a more balanced gut microbiome. We observed a slight increase in the abundance of Bacillota. Members of the phylum Bacillota, including Lactobacillaceae, Lachnospiraceae, and Ruminococcaceae, can break down complex polysaccharides through hydrolytic processes and generate SCFAs.^[24]^ There have been various reports on the minor effects of probiotics on the gut microbiome of healthy individuals.^[25,26]^ Majeed et al^[27]^ reported a significant positive change in the microbial phyla and species, which may positively affect healthy subjects. The combination of the three probiotic strains was found to influence both circulating interleukin-6 levels and gut microbiome composition.^[28]^ In healthy children, consumption of probiotic drinks is linked to notable changes in the gut microbiome, particularly in the populations of *Lactobacillus* spp. and *Bifidobacterium* spp.^[29]^ A study by Toscano et al^[30]^ also demonstrated a decrease in the Pseudomonadota phylum following the ingestion of *Lactobacillus rhamnosus* and *Bifidobacterium longum* over a one-month period.

SCFAs are metabolites produced through the microbial fermentation of dietary fibers.^[31]^ Dysbiosis of the gut microbiota results in decreased SCFA production, and insufficient SCFA concentration in the body may contribute to various pathogenic disorders.^[32]^ The results of the SCFA analysis revealed that following probiotic administration, there was a subtle increase in the concentrations of acetic acid and propionic acid, whereas butyric acid concentrations remained relatively constant. This observation aligns with a study conducted on diverse age groups treated with *Lactobacillus plantarum,* which reported significant increases in acetic acid and propionic acid levels, whereas butyric acid levels exhibited no significant change.^[33]^ This suggests that probiotics positively influenced SCFA production. In children with normal weight, a substantial increase from baseline levels was observed in both the overall short-chain fatty acid and propionic acid levels, following the administration of a probiotic-based beverage.^[29]^ Our previous study on the gut-brain axis in an animal model showed that *B. coagulans* BCP92 modulates SCFA levels, such as acetic and butyric acids.^[34]^

Safety evaluation indicated that no adverse reactions were observed or reported during the study. Additionally, all blood test parameters remained within normal ranges, indicating that the product was safe for consumption. These results corroborate those of other published safety studies.^[16,35]^ A limitation of the present analysis is that multiple comparisons were performed across several biochemical and hematological parameters and assessment visits without adjustment for multiplicity. Therefore, some nominally significant findings may represent chance findings due to multiple testing. However, these findings were not considered clinically significant and did not indicate any clinically relevant safety concern.

## 5. Conclusion

Recent randomized controlled trials involving healthy individuals have demonstrated minor alterations in gut microbiome composition and a slight increase in SCFAs, specifically acetate and propionate. These results indicate that the consumption of probiotics may positively affect the gut microbiome and enhance SCFA production. Additionally, the results confirmed that *B. coagulans* BCP92 is a safe probiotic, maintaining the tested blood parameters within the normal range and exhibiting no adverse events. It should be emphasized that this study spanned only a 42-day period. This duration implies that extended probiotic use might lead to more substantial improvements in both gut microbiome composition and SCFA levels. Such changes could potentially contribute to maintaining eubiosis through modulation of the gut microbiome.

## Acknowledgments

The authors acknowledge the facilities provided by Cliantha Research, Ahmedabad, for conducting this research.

## Author contributions

**Conceptualization:** Sohel S. Shaikh, Farhana Malek.

**Formal analysis:** Sohel S. Shaikh, Farhana Malek.

**Investigation:** Sohel S. Shaikh, Farhana Malek.

**Methodology:** Sohel S. Shaikh.

**Project administration:** Sohel S. Shaikh.

**Resources:** Sohel S. Shaikh.

**Supervision:** Sohel S. Shaikh.

**Validation:** Sohel S. Shaikh.

**Writing – review & editing:** Sohel S. Shaikh, Farhana Malek.

**Writing – original draft:** Farhana Malek.


