# Supplementary material for: Healthy subjects gut microbiome modulation by Bacillus coagulans BCP92: A randomized, double-blind, placebo-controlled clinical trial
Source: Medicine (Baltimore). 2026 Aug 28;105(35):e50435. doi: 10.1097/MD.0000000000050435 (PMC13529129; doi:10.1097/MD.0000000000050435)
Supplement: Supplementary file 1 [file medi-105-e50435-s001.docx]

**Supplementary Table 1b: Microscopic Examination of Urine Tests (Frequency and Percentage)**

| **Visit** | **Test name** | **Result** | **IP (N=23)** | **Placebo (N=20)** | **Overall (N=43)** |
| --- | --- | --- | --- | --- | --- |
| **Baseline** | **Amorphous Material** | Absent | 23 (100%) | 20 (100%) | 43 (100%) |
|  | **Bacteria** | Absent | 23 (100%) | 20 (100%) | 43 (100%) |
|  | **Casts** | Absent | 23 (100%) | 20 (100%) | 43 (100%) |
|  | **Crystals** | Absent | 23 (100%) | 20 (100%) | 43 (100%) |
|  | **Epithelial Cell** | 0-1 | 23 (100%) | 20 (100%) | 43 (100%) |
|  | **Monilia** | Absent | 23 (100%) | 20 (100%) | 43 (100%) |
|  | **Pus Cells (WBCs)** | 0-1 | 23 (100%) | 20 (100%) | 43 (100%) |
|  | **Red Blood Cells** | 0-1 | 1 (4.35%) | 0 (0%) | 1 (2.33%) |
|  |  | Nil | 22 (95.65%) | 20 (100%) | 42 (97.67%) |
|  | **Trichomonas** | Absent | 23 (100%) | 20 (100%) | 43 (100%) |
| **End of the Study** | **Amorphous Material** | Absent | 23 (100%) | 20 (100%) | 43 (100%) |
|  | **Bacteria** | Absent | 23 (100%) | 20 (100%) | 43 (100%) |
|  | **Casts** | Absent | 23 (100%) | 20 (100%) | 43 (100%) |
|  | **Crystals** | Absent | 23 (100%) | 20 (100%) | 43 (100%) |
|  | **Epithelial Cell** | 0-1 | 17 (73.91%) | 13 (65.00%) | 30 (69.77%) |
|  |  | 1-2 | 6 (26.09%) | 7 (35.00%) | 13 (30.23%) |
|  | **Monilia** | Absent | 23 (100%) | 20 (100%) | 43 (100%) |
|  | **Pus Cells (WBCs)** | 0-1 | 20 (86.96%) | 16 (80.00%) | 36 (83.72%) |
|  |  | 1-2 | 3 (13.04%) | 4 (20.00%) | 7 (16.28%) |
|  | **Red Blood Cells** | 0-1 | 1 (4.35%) | 1 (5.00%) | 2 (4.65%) |
|  |  | Nil | 21 (91.30%) | 16 (80.00%) | 37 (86.05%) |
|  |  | 1-2 | 1 (4.35%) | 3 (15.00%) | 4 (9.30%) |
|  | **Trichomonas** | Absent | 23 (100%) | 20 (100%) | 43 (100%) |
